# *Cryptosporidium parvum*, a potential cause of colic adenocarcinoma

**DOI:** 10.1186/1750-9378-2-22

**Published:** 2007-11-21

**Authors:** Gabriela Certad, Tramy Ngouanesavanh, Karine Guyot, Nausicaa Gantois, Thierry Chassat, Anthony Mouray, Laurence Fleurisse, Anthony Pinon, Jean-Charles Cailliez, Eduardo Dei-Cas, Colette Creusy

**Affiliations:** 1 Ecologie du Parasitisme (EA3609 Université de Lille 2), IFR 142, Institut Pasteur de Lille, Lille, France; 2 Cátedra de Parasitología, Escuela de Medicina "José María Vargas", Universidad Central de Venezuela (UCV), Caracas, Venezuela; 3 Plateau d'Expérimentation Animale, Institut Pasteur de Lille, France; 4 Service d'Anatomie et de Cytologie Pathologiques, Groupe Hospitalier de l'Université Catholique de Lille, France; 5 Unité de Sécurité Microbiologique, Institut Pasteur de Lille, France; 6 Laboratoire Environnement et Santé, Université Catholique de Lille, France; 7 Parasitologie-Mycologie, Centre Hospitalier Régional et Universitaire de Lille, Université de Lille 2, France

## Abstract

**Background:**

Cryptosporidiosis represents a major public health problem. This infection has been reported worldwide as a frequent cause of diarrhoea. Particularly, it remains a clinically significant opportunistic infection among immunocompromised patients, causing potentially life-threatening diarrhoea in HIV-infected persons. However, the understanding about different aspects of this infection such as invasion, transmission and pathogenesis is problematic. Additionally, it has been difficult to find suitable animal models for propagation of this parasite. Efforts are needed to develop reproducible animal models allowing both the routine passage of different species and approaching unclear aspects of *Cryptosporidium *infection, especially in the pathophysiology field.

**Results:**

We developed a model using adult severe combined immunodeficiency (SCID) mice inoculated with *Cryptosporidium parvum *or *Cryptosporidium muris *while treated or not with Dexamethasone (Dex) in order to investigate divergences in prepatent period, oocyst shedding or clinical and histopathological manifestations. *C. muris*-infected mice showed high levels of oocysts excretion, whatever the chemical immunosuppression status. Pre-patent periods were 11 days and 9.7 days in average in Dex treated and untreated mice, respectively. Parasite infection was restricted to the stomach, and had a clear preferential colonization for fundic area in both groups. Among *C. parvum*-infected mice, Dex-treated SCID mice became chronic shedders with a prepatent period of 6.2 days in average. *C. parvum*-inoculated mice treated with Dex developed glandular cystic polyps with areas of intraepithelial neoplasia, and also with the presence of intramucosal adenocarcinoma.

**Conclusion:**

For the first time *C. parvum *is associated with the formation of polyps and adenocarcinoma lesions in the gut of Dex-treated SCID mice. Additionally, we have developed a model to compare chronic *muris *and *parvum *cryptosporidiosis using SCID mice treated with corticoids. This reproducible model has facilitated the evaluation of clinical signs, oocyst shedding, location of the infection, pathogenicity, and histopathological changes in the gastrointestinal tract, indicating divergent effects of Dex according to *Cryptosporidium *species causing infection.

## Background

Cryptosporidiosis represents a major public health problem. This infection, caused by protozoa of the genus *Cryptosporidium*, has been reported worldwide as a frequent cause of diarrhoea, and its prevalence varies according to different regions [1]. In developed countries, massive *Cryptosporidium *foodborne and waterborne outbreaks have been reported. In developing countries, *Cryptosporidium *affects mostly children under five [2]. Furthermore, cryptosporidiosis remains a clinically significant opportunistic infection in immunocompromised patients, causing potentially life-threatening diarrhoea, especially in those HIV-infected without access to highly active antiretroviral therapy (HAART) [3]. Additionally, these parasites not only infect humans, but also cause morbidity in farm animals, leading to economic losses [4]. Most *Cryptosporidium *species infect the epithelium of the gut but in severe infections, dissemination can occur to extra-intestinal sites [5]. Infection of the intestinal cells can result in blunting of the intestinal villi, crypt hyperplasia and inflammation. Epithelial cell apoptosis due to this parasite has also been described [6,7].

Molecular techniques have been developed to differentiate this parasite at the species and genotype levels, showing that there are at least 16 different species [1], and several methods have been used to study and characterize different parasite strains. However, the understanding about invasion, transmission, pathogenesis and epidemiology is limited, and no effective drug against this infection is available. Previous studies based on multilocus characterisation of *Cryptosporidium *isolates identified different sub-groups within some *Cryptosporidium *species [8-11]. These groups exhibited different population genetic structures (epidemic clonality and panmixia) that could be correlated to defined phenotypes.

To contribute to the comprehension of the dynamics of infection and to investigate biological divergence between different populations of *Cryptosporidium*, tissue culture and animal models have been used. It has been difficult to find suitable models for cryptosporidiosis, as most mammalians are susceptible to infection only as newborns [12]. However, some *Cryptosporidium *species can be propagated in either chemically or genetically immunosuppressed mice. Particularly, adult mice with congenital mutations such as *nu/nu*, *scid*, *bg/nu, xid *or mice with steroid induced immunosuppression are susceptible to infection [12-17]. Efforts are still needed to develop reproducible animal models allowing both the routine passage of different species and approaching unclear aspects of *Cryptosporidium *infection, especially in the pathophysiology field.

We have developed a model to compare chronic *muris *and *parvum *cryptosporidiosis using SCID mice treated with corticoids in order to evaluate clinical signs, location of the infection, pathogenicity, oocyst shedding and histopathological changes in the gastrointestinal tract. This model was chosen for the following reasons: i) Previous studies have shown that host cell immunity during cryptosporidiosis is mediated by both Th1 and Th2 response [18,19], thus SCID mice are more susceptible to the infection and to develop a chronic disease due to their defect in T and B lymphocytes [20]. ii) It has been found that during cryptosporidiosis there is an IFNγ mucosal response with increased levels of Il15 [21]. IFNγ-induced enterocyte resistance against *C. parvum *has been reported [22]. Furthermore, glucocorticoids are known to have an effect on the priming of the innate immune response [23], and could suppress IFNγ-regulated gene expression [24]. Consequently, dexamethasone, a synthetic glucocorticosteroid, could be used to alter this innate immune response. iii) This model of SCID mice treated with steroids has been proven to be successful for the development of *Pneumocystis*, another opportunistic agent [25].

## Results

The pathological damage due to *C. muris *or *C. parvum *infection was studied in SCID mice treated or not with Dex. Main data related to infected mice included in the study are shown in Table 1.

**Table 1:** Experimental infection of Dexamethasone-treated or untreated SCID mice infected with *C. parvum *or *C. muris*: Main clinical and histopathological data

Group^a^	Mouse N°	Day of euthanasia (post-infection)	Oocysts/mg faeces at euthanasia	Clinical manifestations	Main histological changes
**P**	1	20	0	Occasional diarrhoea	Undetected
	2	28	0	Lethargy	Undetected
	3	28	1	None	N.D.
	4	84	1	Occasional diarrhoea	Undetected
	5	84	4	Occasional diarrhoea	Undetected
	6	84	0	Occasional diarrhoea	N.D.
**Pdex**	7	20	29	Occasional diarrhoea	Undetected
	8	28	102	Lethargy, ruffled coat	Undetected
	9	28	95	Lethargy, ruffled coat	N.D.
	10	46	1182	Lethargy, ruffled coat	Polyps with areas of low-grade and high-grade intraepithelial neoplasia, and intramucosal adenocarcinoma at the ileocaecal region
	11	62	240	Lethargy, ruffled coat	Polyps with areas of low-grade and high-grade intraepithelial neoplasia, and intramucosal adenocarcinoma at the ileocaecal region
	12	84	695	Lethargy, ruffled coat	Polyps with areas of low-grade and high-grade intraepithelial neoplasia, and intramucosal adenocarcinoma at the ileocaecal region
**M**	13	20	1078	None	Stomach heavily infected mainly at the fundic region; dilated glands; hyperplasia
	14	20	407	None	N.D.
	15	28	742	None	Stomach heavily infected mainly at the fundic glands, hyperplasia
	16	84	4143	None	Stomach heavily infected mainly at the fundic region; dilated glands; hyperplasia
	17	84	4920	None	Stomach heavily infected
	18	84	5828	None	None
**Mdex**	19	20	1538	Occasional diarrhoea	Stomach heavily infected; dilated glands
	20	20	166	Occasional diarrhoea	N.D.
	21	27	1241	Occasional diarrhoea	N.D.
	22	32	4155	Frequent diarrhoea, lethargy, ruffled coat	Stomach heavily infected mainly at the fundic glands; hyperplasia
	23	46	900	Lethargy, ruffled coat	Stomach heavily infected mainly at the fundic glands; hyperplasia
	24	84	2051	None	Stomach heavily infected mainly at the fundic glands; hyperplasia; a little inflammation
**C**	25	20	0	None	Undetected
	26	67	0	Lethargy, ruffled coat	Undetected
	27	84	0	None	Undetected
**Cdex**	28	20	0	None	Undetected
	29	43	0	None	Undetected
	30	84	0	None	Undetected
**Cdex2**	31	54	0	None	Undetected
	32	54	0	None	Undetected
	33	54	0	None	Undetected
	34	54	0	None	Undetected
	35	54	0	None	Undetected
	36	54	0	None	Undetected

^a^Experimental groups were:

**P**: *C. parvum*-infected SCID mice; **PDex**: *C. parvum*-infected Dex-treated SCID mice; **M**: *C. muris*-infected SCID mice; **MDex**: *C. muris*-infected Dex-treated SCID mice; **C**: Not infected SCID mice of control group inoculated with PBS; **CDex**: Not infected Dex-treated SCID mice of control group inoculated with PBS; **CDex2**: Not infected Dex-treated SCID mice that received an inoculum from which oocysts were previously removed by filtration; **N.D**.: Not done (These mice were not included in the histological examination).

**Figure 1:**
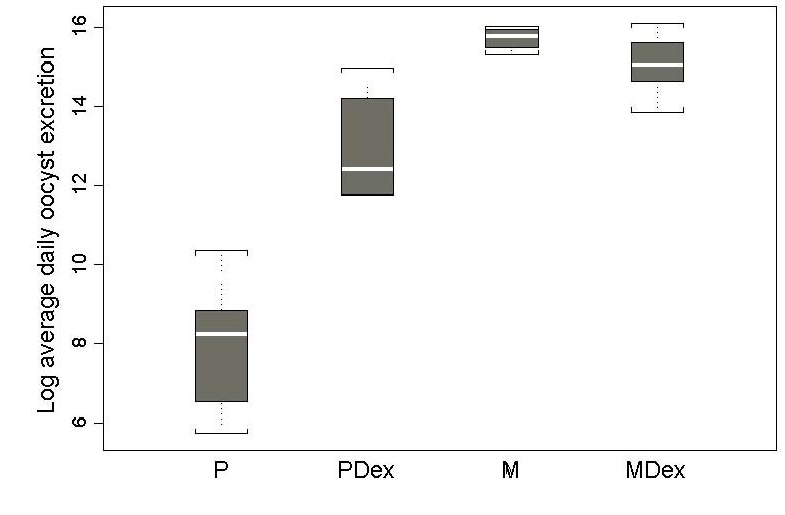
**Napierian logarithm of daily oocyst excretion (oocyst/mg faeces) in different groups of mice**. Experimental groups were: P: *C. parvum*-infected SCID mice; PDex: *C. parvum*-infected Dex-treated SCID mice; M: *C. muris*-infected SCID mice; MDex: *C. muris*-infected Dex-treated SCID mice. Each box represents the middle half of data, the white line being the median. Whiskers represent the extreme values within 1.5 times the box height.

Dex induced a significant body weight loss in both *Cryptosporidium *infected and uninfected SCID mice (*P *< 0.001). In contrast, no significant body weight change was associated with parasite infection. Once infected with *C. muris*, mice from groups M and MDex became chronic shedders and produced high numbers of oocysts without marked differences between the groups. On the other hand, in animals inoculated with *C. parvum*, the oocyst excretion was sporadic and limited in P mice, and high and chronic in PDex mice, with a statistically significant difference (*P *= 0.002). Figure 1 shows the average of oocyst excretion in different groups of mice. Analysis of variance showed that the administration of Dex, *Cryptosporidium *species, and the interaction of these factors significantly influenced oocyst excretion (all the *P *< 0.001). The level of oocyst excretion was higher in mice infected with *C. muris *than in those infected with *C. parvum*.

**Figure 2:**
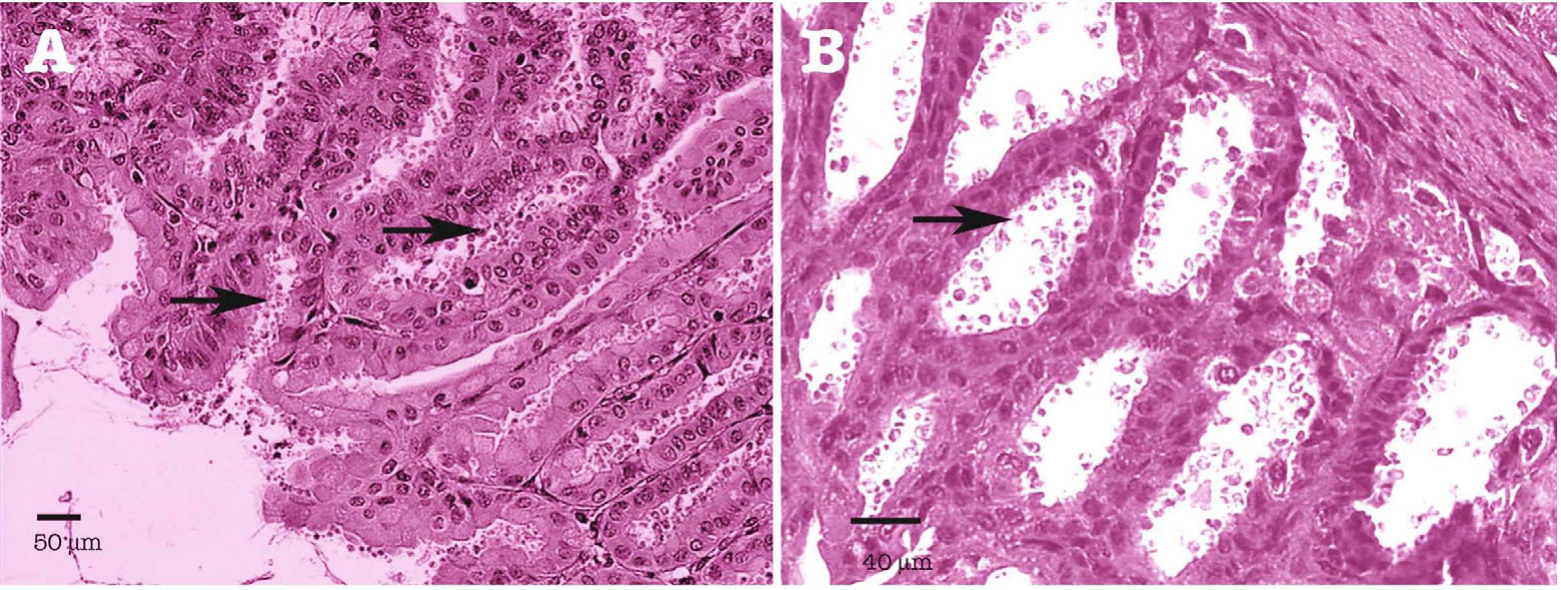
**Experimental *Cryptosporidium muris *infection of SCID mice**. (A) Stomach section from a Dex-untreated SCID mouse euthanatized at day 84 post-infection; (B) Stomach section from a Dex-treated SCID mouse euthanatized at day 46 post-infection. In both cases, gastric glands are filled with numerous parasites at different developmental stages (arrows). No signs of inflammation. Hematoxylin & Eosin staining.

Pre-patent period ranged from 6 to 11 days (9.7 days in average) in M mice and it was 11 days for all MDex mice. Geometric means of oocyst excretion before sacrifice were 1839 and 1159 oocysts/mg faeces respectively (Table 1). At histological level, no difference between mice of groups M or MDex was observed. *C. muris *localization was restricted to the stomach, with no extra-gastric dissemination. Different stages of the parasite life cycle were present. Stomachs of all mice euthanatized at different dates, from day 20 to day 84 post-infection, were heavily infected mainly in fundic mucosae (Figure 2). Fundic gastric glands were dilated and covered by a flattened epithelium. Oxyntic cells were less numerous than in control mice and preferentially visible in the deeper area. Fundic mucosa was twice thinner than in control mice while pyloric mucosa was similar to that in uninfected control mice. A moderate inflammatory infiltrate, made of mononuclear cells and neutrophile polynuclears, was observed in some cases.

**Figure 3:**
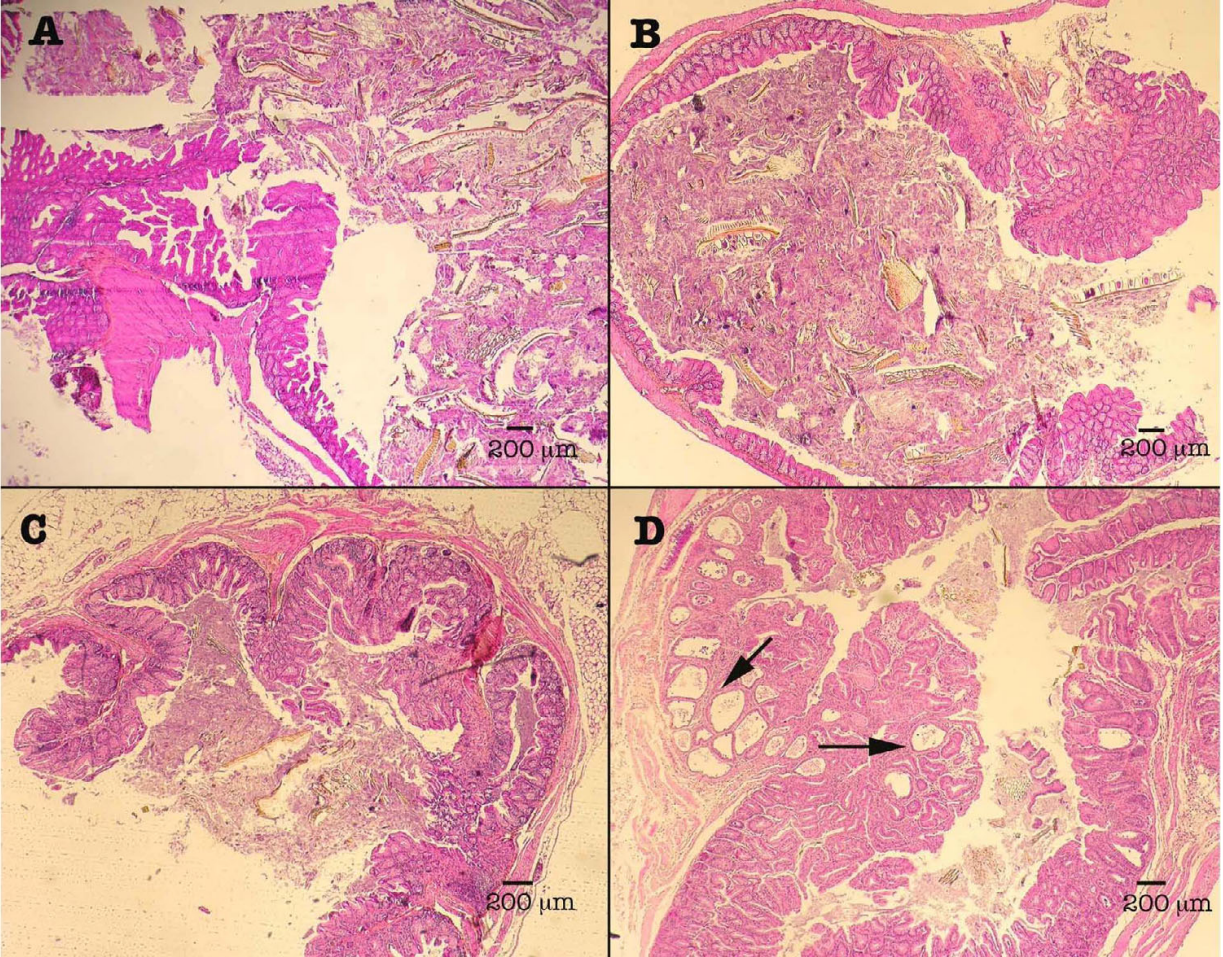
**Ileocaecal regions of mice from different groups**. Normal caecum of (A) Uninfected mouse administered with dexamethasone (CDex group), (B) *C. muris*-infected Dex-treated mouse (MD group) and (C) *C. parvum*-infected mouse (P group). (D) Projection of polypoid structures with focal cystic dilation (arrows) developing inside the intestinal lumen of a *C. parvum*-infected Dex-treated SCID mouse. Hematoxylin & Eosin staining.

For *C. parvum*-infected SCID mice from group P, geometric mean of oocyst excretion before euthanasia was 1 oocyst/mg faeces. SCID mice from PDex group had a prepatent period ranging from 4 to 11 days (6.2 days in average) and developed a chronic infection. Geometric mean of oocyst excretion before sacrifice was 195 oocysts/mg of faeces for mice from group PDex. Two out of six PDex SCID mice were severely ill and required euthanasia. In mice infected with *C. parvum*, histopathological differences were revealed between Dex-treated and untreated animals. Untreated SCID mice infected with *C. parvum *have neither detectable parasites nor lesions at the histological level (Figure 3) at any time during the course of the study.

**Figure 4:**
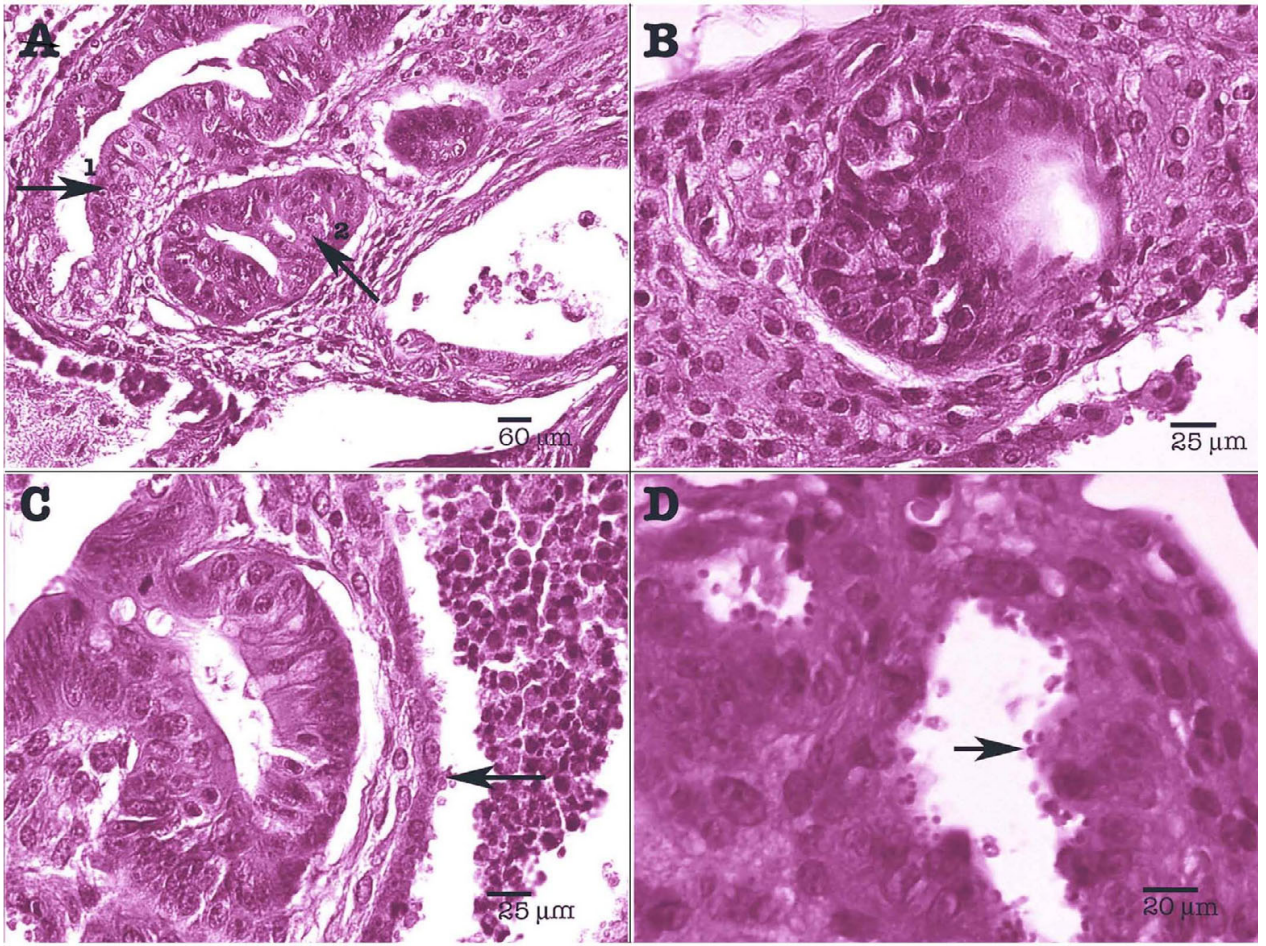
**Experimental *Cryptosporidium parvum *infection of Dex-treated SCID mice: caecal region**. (A) Polyp with areas of high-grade intraepithelial neoplasia (arrow 1) and intramucosal adenocarcinoma (arrow 2); (B) Abnormal nuclear changes consisting of prominent nucleoli and irregularly scattered chromatin; (C) Highly irregular glands, areas of loss of glandular differentiation. Presence of numerous parasites (arrow); (D) Presence of numerous parasites at different developmental stages in the intestinal epithelium (arrow). Hematoxylin & Eosin staining.

In Dex-treated SCID mice infected with *C. parvum*, parasite localization was restricted to the gastrointestinal tract, mainly at the caecal region (Figure 3). Three out of five (60%) histologically examined PDex SCID mice presented in the ileocaecal region polypoid, sessile, adenomatous masses, measuring approximately 2.5 mm in diameter. They appeared as closely packed, branching sometimes dilated tubular structures, separated by normal or inflammatory lamina propria. Focal cystic dilation was observed. Noticeably some tubules were covered by a low grade or high grade dysplastic epithelium, which showed mucin depletion and nuclear stratification. In some areas, architectural distortion was associated with marked cellular atypias (Figure 4). Epithelial cells presented a loss of their normal polarity. In addition, abnormal nuclear changes consisting of prominent nucleoli and irregularly scattered chromatin were observed (Figure 4). These mucosal changes were suggestive of intraepithelial neoplasia of low or high grade. In some areas, major cellular atypias, with foci of merged glands, typical of intramucosal adenocarcinoma, invasive into lamina propria, were found (Figures 4A and 4B).

**Figure 5:**
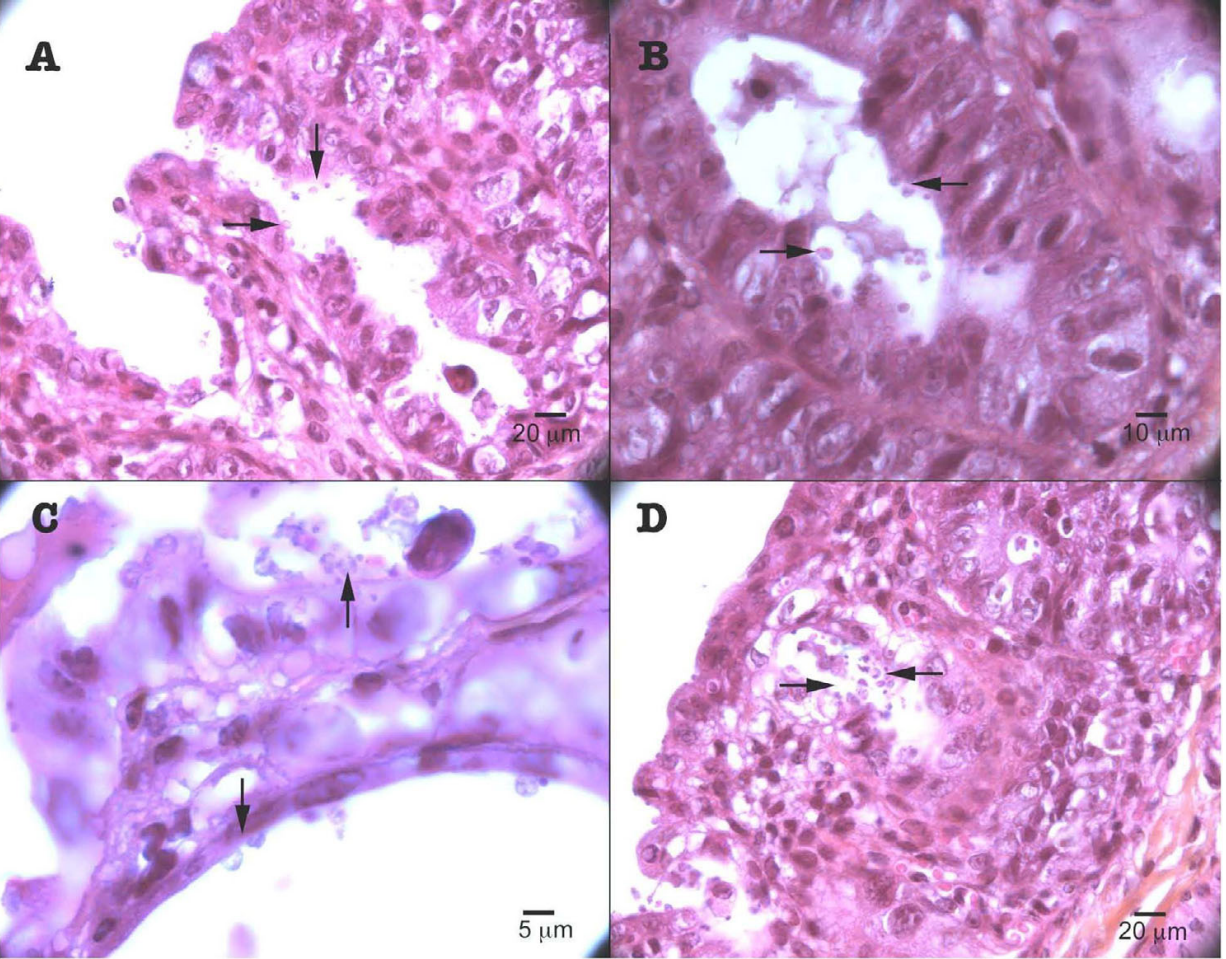
**Experimental *Cryptosporidium parvum *infection of Dex-treated SCID mice**. Low (A, D) and high magnification (B, C) of the caecal region showing the presence of abundant parasites (arrow), and high degree dysplasia (arrow). Hematoxylin & Eosin staining.

These major histological changes were earliest observed in one mouse euthanatized at day 46 post-infection and were found in all PDex mice necropsied after (Table 1). They were always associated with the presence of *C. parvum *organisms (Figures 4 and 5). In contrast, no parasite was detected in the stomach, duodenum, jejunum, hepatic biliary system or pancreas. The possibility that biotic (e.g. virus) or abiotic factors present in the inoculum could be responsible for these lesions was discarded by administering Dex-treated SCID mice with a filtered inoculum (i.e. without *Cryptosporidium *oocysts) (group Cdex2). Neither parasites in the faeces or tissues nor lesions were detected in these mice at any time of the experience.

## Discussion

We developed a new animal model that allows a good propagation of two different species of *Cryptosporidium*. In this study, adult SCID mice treated with Dex became chronically infected with 10^5 ^*C. parvum *or *C. muris *oocysts, and had a significant oocyst shedding during all the course of the experiments. Furthermore, this model was useful to compare *C. muris *and *C. parvum *infections at clinical, histopathological and parasitological levels.

In this study, variations in the expression of the disease in terms of either *Cryptosporidium *species or Dex administration were shown. Amounts of oocysts discharged seemed slightly lower in MDex than in M mice but this difference was not statistically significant. However, it appeared that the aggravation of the immunosuppression status did not lead to an increase in the severity of *C. muris *infection. Miller *et al*. previously reported that immunosuppressed mice were as or less susceptible to *C. muris *than immunocompetent mice [26]. However, this conclusion can hardly be extrapolated to all *Cryptosporidium *species, as long as in our experiments, the oocyst shedding was markedly and significantly higher in PDex than in P group. Further studies are required to determine minimal infectious dose, ID_50 _(infectious dose to 50 percent of exposed individuals) and other data for each species and according to the immunosuppression degree. Interestingly, data from very recent experiments with *C. parvum *confirmed the results of the present work (data not shown). The Dex-treated SCID mouse model revealed therefore to be reproducible.

*C. muris *infection caused damage to the gastric mucosa of both mice from M and MDex groups. But though M mice were less ill than MDex mice, there was no marked histological difference between them. Dilated, hypertrophied and highly parasitized gastric glands without an extra gastric dissemination of parasites were the main histopathological changes. Additionally, a little inflammatory response was observed. Other authors have reported similar findings in relation with *C. muris *gastric infection [12,27,28]. However, to our knowledge, this is the first report of a clear preferential localization of the parasite colonization at the fundic level of the stomach, where acid secreting glands are numerous [29]. This could suggest a favourable influence of lower gastric pH on the growth of *C. muris*.

On the other hand, several studies using animal models have described histological changes in the intestinal epithelium due to *C. parvum *infection, such as villous atrophy and crypt hyperplasia in the lower small intestine [13] or in the caecum and the colon [12], cryptic hyperplasia with abscesation of crypts in the large intestine [13], stunting and fusion of villi, replacement of enterocytes by immature cells and eosinophilia of lamina propria [30], small and large intestine mucosa severely damaged with villous contraction and little or absent epithelial layer [31]. Another report described an association between *Cryptosporidium *sp. and aural-pharyngeal polyps in iguanas. These polyps were pedunculated masses composed of glandular cystic structures lined by hyperplastic cuboidal to columnar epithelium, containing numerous parasites along the apical surfaces of the epithelial cells [32]. Nevertheless, none of these studies have described the presence of carcinoma lesions associated to cryptosporidiosis. These lesions were first observed unexpectedly after 46 days post-infection, when the infection had become chronic, and were found only in *C. parvum *infected SCID mice treated with Dex. These findings were recorded using an inoculum relatively low (10^5 ^oocysts) in comparison with the higher infectious doses, between 10^6 ^and 10^7^, used by others [12,33].

Several observations in our study suggest that combination of *C. parvum *with Dex administration is involved in the generation of these significant histological changes. Indeed, Dex seemed to be a critical factor to the development of intraepithelial adenocarcinoma in *C. parvum*-infected SCID mice. Dex potentially alters the innate immunity in *C. parvum *infected animals [23,24]. The higher oocyst shedding found in mice presenting these neoplasic lesions seems to confirm this assumption. Additionally, this kind of lesion was found neither in Dex-untreated *C parvum*-infected animals with a low oocyst excretion during the course of the study, nor in the control Dex-treated non-infected group. A possible contamination of the inoculum with a virus or other agent potentially responsible for these neoplasic lesions was also discarded. Interestingly, *C. muris *was not able to induce this type of epithelial changes and the reasons of this different expression of the disease are unclear. It has been reported previously a variability in pathogenicity for different hosts between different *Cryptosporidium *species and types, suggesting the existence of specific virulence factors among species and isolates [34]. Further studies should be done to clarify this difference in pathogenicity. It is important to mention that these colonic neoplasic lesions are not listed among the typical background diseases due to the SCID mice genetic defect [35].

To our knowledge, this is the first time that *C. parvum *is associated with cancerogenesis. There is one case report that described cryptosporidiosis of the biliary tract clinically mimicking a pancreatic cancer in an AIDS patient [36]. However, biopsy of the gallbladder revealed cryptosporidiosis causing inflammation of the biliary tract and ruled out the diagnosis of neoplasia [36]. Furthermore, a recent epidemiological study in Poland reported a high frequency of cryptosporidiosis in patients with colorectal cancer [37]. These data strengthen the interest of our experimental observations.

Several infectious agents, including parasites, have been linked to oncogenesis in humans. Some of these associations are strong. Particularly, *Schistosoma hematobium *has been described as a definitive cause of urinary bladder cancer by the International Agency for Research in Cancer. Also, a proportion of cholangiocarcinoma of the liver worldwide is attributable to Opisthorchiidae liver flukes [38]. Other associations still speculative with some protozoa were suggested on the basis of epidemiological observations, such as *Trichomona vaginalis *and cervical cancer, or *Toxoplasma gondii *and tumors [39]. As well, a microbial pathogen such as *Helicobacter pylori *has been classified as oncogenic for humans due to its strong epidemiological association with carcinoma of stomach and gastric lymphoma [38,40].

Because *C. parvum *is an opportunistic agent that causes significant morbidity and mortality in immunocompromised patients, it is possible that individuals infected with this parasite may have a higher risk of developing colorectal malignancies, especially when immunosuppression is more severe. In a retrospective study that shows the incidence and clinical course of colorectal malignancies in HIV/AIDS patients, adenocarcinoma was the type of cancer most frequently found among them. These patients did not have familial antecedents of intestinal malignancies or other risk factors, and developed tumors at earlier ages in comparison to immunocompetent persons [41]. Unfortunately, no data about gastro-intestinal parasites in these patients were given in the study [41]. More studies have to be done in humans to evaluate cryptosporidiosis as a possible risk factor of colorectal cancer.

Epidemiological studies have shown that environmental factors may play a role in colon cancer susceptibility [42]. The association between chronic inflammation and cancer is also well known [43]. Particularly, colon cancer has also been associated to inflammatory bowel disease. In the latter case, cancer develops after sustained gut inflammation. However, in the present work *C. parvum *infection was associated with only mild inflammation of the intestine as reported also by others [12].

Taking into consideration that the observations described herein were replicated in a second experiment (unpublished data), it can be concluded that *C. parvum *is able to induce the formation of polyps and carcinogenic lesions in the gut of Dex-treated SCID mice. However, more work has to be done to elucidate this interesting association and to further understand cryptosporidiosis pathogenesis.

## Conclusion

In summary, for the first time *C. parvum *was associated with the generation of polyps and *in situ *adenocarcinoma in the gut of Dex-treated SCID mice. Additionally, we have developed a model to compare chronic *muris *and *parvum *cryptosporidiosis using SCID mice treated with corticoids. This reproducible model has allowed the evaluation of clinical impact, oocyst shedding, location of the infections, pathogenic power, and histopathological changes indicating divergent effects of Dex according to *Cryptosporidium *species.

## Methods

### *Cryptosporidium *oocysts

*C. parvum *IOWA and *C. muris *RN66 oocysts were purchased from Waterborne™, Inc. (New Orleans, Louisiana) and stored in shipping medium (Phosphate-buffered saline (PBS) with penicillin, streptomycin, gentamycin, amphotericin B and 0.01% Tween 20) at 4°C until use. Oocyst viability before inoculation was determined by a trypsin-taurocholate excystation test [44] and absence of germs was assured by testing the oocyst suspensions on Plate Count Agar and on Sabouraud plates.

### Animals

Nine week-old male and female CB17-SCID mice were obtained from a colony bred at the Pasteur Institute of Lille (France), and maintained under aseptic conditions in an isolator, with standard laboratory food for experimental mice and water *ad libitum*. All cages, food, water and bedding were sterilized before use. Faecal pellets were collected from all mice before inoculation to ascertain the absence of pre-existing *Cryptosporidium *infection. The conditions for the care of laboratory animals stipulated in European guidelines (Council directives on the protection of animals for experimental and other scientific purposes. J. Off. Communautés Européennes, 86/609/EEC, 1986, December 18^th^, L358) were followed.

### Immunosuppression

When needed (see the next section), SCID mice were chemically immunosuppressed by administering 4 mg/L of dexamethasone sodium phosphate (Dex) (Merck, Lyon, France) via the drinking water. Immunosuppression was started two weeks prior to inoculation and was maintained during the whole experimentation. Dex-added water was replaced three times a week.

### Experimental design

Twenty-four SCID mice, housed individually (one-mouse-cage) in capped cages, were randomly divided into four groups: P, M, PDex and MDex, described hereinafter. Six SCID mice, housed by three, constituted two control groups, C and CDex. Oral inoculation was done with 10^5 ^oocysts in 200 μl of PBS. Six mice were infected with *C. parvum *oocysts (group P) and six others with *C. muris *oocysts (group M). Under the effect of Dex immunosuppression, six mice were infected with *C. parvum *oocysts (group PDex) and six others with *C. muris *oocysts (group MDex). Mice of control groups were inoculated only with PBS, without (group C) or with (group CDex) Dex administration. A further control group was constituted by six SCID mice that received an inoculum from which oocysts were previously removed by filtration with a Nanosep MF tube with a 0.45 μm pore size membrane (group CDex2).

### Quantification of the oocyst shedding

To evaluate the intensity of oocyst shedding over the course of *Cryptosporidium *infection, freshly excreted faecal pellets were collected three times a week from each mouse and suspended in MilliQ water. Oocysts were detected and numbered after staining faecal smears with modified Ziehl-Neelsen stain [45].

### Statistical analysis

Analyses of data were performed with the statistical software S-PLUS 2000 (MathSoft, Seattle, WA, USA). For statistical analyse purposes, logarithmic transformations of oocyst excretion values were used. Wilcoxon rank sum tests were used to compare body weights or oocyst excretion. An analysis of variance was conducted to account for the effects of relevant factors and their interactions on average daily excretion of oocysts. An average amount of oocysts excreted per day was estimated for each mouse from all the unitary values, and was represented as a function of the mouse group in a Box & Whiskers Plot.

### Histological examination

Periodically or when signs of imminent death appeared, mice were euthanatized by a sodium pentobarbital (Ceva, Libourne, France) intra-cardiac injection. The liver, stomach and pancreas, a section of the duodenum, 3 sections of the jejunum, the caecum, and 3 sections of the colon were removed. Tissues were fixed in 10% neutral formalin and embedded in paraffin. Histological sections were stained with hematoxylin and eosin, and examined microscopically for the detection of *Cryptosporidium *organisms and/or histological modifications of the host tissue. Pathological changes found in the mouse caecum were classified according to the Vienna classification of tumors of the digestive system [46,47].

## Competing interests

The author(s) declare that they have no competing interests.

## Authors' contributions

GC & TN have equally contributed to this work. They participated in the conception and design of the study, carried out the experiments and drafted the manuscript. KG participated in the design of the experiments. NG, TC, AM participated in the performance of animal experiments. LF prepared the histological cuts. AP carried out the statistical analysis. JCC participated in the design of the study. ED participated in the design and coordination of the study and helped to draft the manuscript. CC carried out the pathological study and helped to draft the manuscript. All authors read and approved the final manuscript.

## References

[B1] Caccio SM, Pozio E (2006). Advances in the epidemiology, diagnosis and treatment of cryptosporidiosis. Expert Rev Anti Infect Ther.

[B2] Xiao L, Fayer R, Ryan U, Upton SJ (2004). Cryptosporidium taxonomy: recent advances and implications for public health. Clin Microbiol Rev.

[B3] Pozio E, Morales MA (2005). The impact of HIV-protease inhibitors on opportunistic parasites. Trends Parasitol.

[B4] Sunnotel O, Lowery CJ, Moore JE, Dooley JS, Xiao L, Millar BC, Rooney PJ, Snelling WJ (2006). Cryptosporidium. Lett Appl Microbiol.

[B5] Lopez-Velez R, Tarazona R, Garcia Camacho A, Gomez-Mampaso E, Guerrero A, Moreira V, Villanueva R (1995). Intestinal and extraintestinal cryptosporidiosis in AIDS patients. Eur J Clin Microbiol Infect Dis.

[B6] Chen XM, Levine SA, Splinter PL, Tietz PS, Ganong AL, Jobin C, Gores GJ, Paya CV, LaRusso NF (2001). Cryptosporidium parvum activates nuclear factor kappaB in biliary epithelia preventing epithelial cell apoptosis. Gastroenterology.

[B7] Mele R, Gomez Morales MA, Tosini F, Pozio E (2004). Cryptosporidium parvum at different developmental stages modulates host cell apoptosis in vitro. Infect Immun.

[B8] Mallon M, MacLeod A, Wastling J, Smith H, Reilly B, Tait A (2003). Population structures and the role of genetic exchange in the zoonotic pathogen Cryptosporidium parvum. J Mol Evol.

[B9] Alves M, Matos O, Antunes F (2003). Microsatellite analysis of Cryptosporidium hominis and C. parvum in Portugal: a preliminary study. J Eukaryot Microbiol.

[B10] Tanriverdi S, Markovics A, Arslan MO, Itik A, Shkap V, Widmer G (2006). Emergence of distinct genotypes of Cryptosporidium parvum in structured host populations. Appl Environ Microbiol.

[B11] Ngouanesavanh T, Guyot K, Certad G, Fichoux YL, Chartier C, Verdier RI, Cailliez JC, Camus D, Dei-Cas E, Banuls AL (2006). Cryptosporidium Population Genetics: Evidence of Clonality in Isolates from France and Haiti. J Eukaryot Microbiol.

[B12] McDonald V, Deer R, Uni S, Iseki M, Bancroft GJ (1992). Immune responses to Cryptosporidium muris and Cryptosporidium parvum in adult immunocompetent or immunocompromised (nude and SCID) mice. Infect Immun.

[B13] Heine J, Moon HW, Woodmansee DB (1984). Persistent Cryptosporidium infection in congenitally athymic (nude) mice. Infect Immun.

[B14] Iseki M, Maekawa T, Moriya K, Uni S, Takada S (1989). Infectivity of Cryptosporidium muris (strain RN 66) in various laboratory animals. Parasitol Res.

[B15] Mead JR, Arrowood MJ, Sidwell RW, Healey MC (1991). Chronic Cryptosporidium parvum infections in congenitally immunodeficient SCID and nude mice. J Infect Dis.

[B16] Petry F, Robinson HA, McDonald V (1995). Murine infection model for maintenance and amplification of Cryptosporidium parvum oocysts. J Clin Microbiol.

[B17] Okhuysen PC, Rich SM, Chappell CL, Grimes KA, Widmer G, Feng X, Tzipori S (2002). Infectivity of a Cryptosporidium parvum isolate of cervine origin for healthy adults and interferon-g knockout mice. J Infect Dis.

[B18] Singh I, Theodos C, Li W, Tzipori S (2005). Kinetics of Cryptosporidium parvum-specific cytokine responses in healing and nonhealing murine models of C. parvum infection. Parasitol Res.

[B19] Riggs MW (2002). Recent advances in cryptosporidiosis: the immune response. Microbes Infect.

[B20] Seydel KB, Stanley SL (1996). SCID mice and the study of parasitic disease. Clin Microbiol Rev.

[B21] Robinson P, Okhuysen PC, Chappell CL, Lewis DE, Shahab I, Lahoti S, White AC (2001). Expression of IL-15 and IL-4 in IFN-gamma-independent control of experimental human Cryptosporidium parvum infection. Cytokine.

[B22] Pollok RC, Farthing MJ, Bajaj-Elliott M, Sanderson IR, McDonald V (2001). Interferon gamma induces enterocyte resistance against infection by the intracellular pathogen Cryptosporidium parvum. Gastroenterology.

[B23] Franchimont D (2004). Overview of the actions of glucocorticoids on the immune response: a good model to characterize new pathways of immunosuppression for new treatment strategies. Ann N Y Acad Sci.

[B24] Stojadinovic O, Lee B, Vouthounis C, Vukelic S, Pastar I, Blumenberg M, Brem H, Tomic-Canic M (2007). Novel genomic effects of glucocorticoids in epidermal keratinocytes: inhibition of apoptosis, interferon-gamma pathway, and wound healing along with promotion of terminal differentiation. J Biol Chem.

[B25] Chabé M, Dei-Cas E, Creusy C, Fleurisse L, Respaldiza N, Camus D, Durand-Joly I (2004). Immunocompetent hosts as a reservoir of Pneumocystis organisms: histological and rt-PCR data demonstrate active replication. Eur J Clin Microbiol Infect Dis.

[B26] Miller TA, Ware MW, Wymer LJ, Schaefer FW (2007). Chemically and genetically immunocompromised mice are not more susceptible than immunocompetent mice to infection with Cryptosporidium muris. Vet Parasitol.

[B27] Taylor MA, Marshall RN, Green JA, Catchpole J (1999). The pathogenesis of experimental infections of Cryptosporidium muris (strain RN 66) in outbred nude mice. Vet Parasitol.

[B28] Miller TA, Schaefer FW (2007). Characterization of a Cryptosporidium muris infection and reinfection in CF-1 mice. Vet Parasitol.

[B29] Soybel DI (2005). Anatomy and physiology of the stomach. Surg Clin North Am.

[B30] Enemark HL, Bille-Hansen V, Lind P, Heegaard PM, Vigre H, Ahrens P, Thamsborg SM (2003). Pathogenicity of Cryptosporidium parvum--evaluation of an animal infection model. Vet Parasitol.

[B31] Tzipori S, Rand W, Griffiths J, Widmer G, Crabb J (1994). Evaluation of an animal model system for cryptosporidiosis: therapeutic efficacy of paromomycin and hyperimmune bovine colostrum-immunoglobulin. Clin Diagn Lab Immunol.

[B32] Uhl EW, Jacobson E, Bartick TE, Micinilio J, Schimdt R (2001). Aural-pharyngeal polyps associated with Cryptosporidium infection in three iguanas (Iguana iguana). Vet Pathol.

[B33] Mead JR, Ilksoy N, You X, Belenkaya Y, Arrowood MJ, Fallon MT, Schinazi RF (1994). Infection dynamics and clinical features of cryptosporidiosis in SCID mice. Infect Immun.

[B34] Okhuysen PC, Chappell CL (2002). Cryptosporidium virulence determinants--are we there yet?. Int J Parasitol.

[B35] Sundberg JP, Shultz LD (1993). The Severe Combined Immunodeficiency (scid) Mutation. JAX NOTES.

[B36] de Souza Ldo R, Rodrigues MA, Morceli J, Kemp R, Mendes RP (2004). Cryptosporidiosis of the biliary tract mimicking pancreatic cancer in an AIDS patient. Rev Soc Bras Med Trop.

[B37] Sulzyc-Bielicka V, Kuzna-Grygiel W, Kolodziejczyk L, Bielicki D, Kladny J, Stepien-Korzonek M, Telatynska-Smieszek B (2007). Cryptosporidiosis in patients with colorectal cancer. J Parasitol.

[B38] Parkin DM (2006). The global health burden of infection-associated cancers in the year 2002. Int J Cancer.

[B39] Khurana S, Dubey ML, Malla N (2005). Association of parasitic infections and cancers. Indian J Med Microbiol.

[B40] Fox JG, Wang TC (2007). Inflammation, atrophy, and gastric cancer. J Clin Invest.

[B41] Yeguez JF, Martinez SA, Sands DR, Sands LR, Hellinger MD (2003). Colorectal malignancies in HIV-positive patients. Am Surg.

[B42] Heyer J, Yang K, Lipkin M, Edelmann W, Kucherlapati R (1999). Mouse models for colorectal cancer. Oncogene.

[B43] Taketo MM (2006). Mouse models of gastrointestinal tumors. Cancer Sci.

[B44] Guyot K, Gireaudot-Liepmann MF, Cabon A, Riveau-Ricard I, Lange M, Delattre JM, Dei-Cas E (2000). Influence Of US Epa 1622 Method Successive Steps On The Viability Of Cryptosporidium Oocysts.. Water Science & Technology.

[B45] Henriksen SA, Pohlenz JF (1981). Staining of cryptosporidia by a modified Ziehl-Neelsen technique. Acta Vet Scand.

[B46] Hamilton SR, Aalfonen LA, eds. (2000). Pathology and Genetics.Tumours of the Digestive System. WHO classification of tumours.

[B47] Stolte M (2003). The new Vienna classification of epithelial neoplasia of the gastrointestinal tract: advantages and disadvantages. Virchows Arch.

